# Panniculitis and pancreatitis: Inflammation and necrotic mechanisms in a patient with alcohol use disorder and alarming suspicion for malignant process

**DOI:** 10.1016/j.radcr.2023.01.058

**Published:** 2023-02-08

**Authors:** Anas Mahmoud, Isaac Soliman, Brooke Kania, Moutaz Ghrewati, Walid Baddoura

**Affiliations:** a Department of Internal Medicine, St. Joseph's University Medical Center, 703 Main St, Paterson, NJ 07503 USA; b Department of Hematology-Oncology, St. Joseph's University Medical Center, 703 Main St, Paterson, NJ 07503 USA; c Department of Gastroenterology, St. Joseph's University Medical Center, 703 Main St, Paterson, NJ 07503 USA

**Keywords:** Panniculitis, Chronic pancreatitis, Osteolytic lesions

## Abstract

Panniculitis is an inflammatory process localized to subcutaneous tissue, with etiologies including infection, malignancy, external insults, enzymatic destructive processes, and inflammatory disorders. The incidence of panniculitis manifesting as necrosis of subcutaneous fat tissue associated with pancreatic diseases is low, which may encompass associated periarthritis with bone necrosis and panniculitis (Pancreatitis, panniculitis and polyarthritis syndrome). Pancreatitis, panniculitis and polyarthritis syndrome is considered to derive from the systemic activity of enzymes within the pancreas, which leads to disturbances within the microcirculatory system, and fat necrosis of medullary bone marrow; however, the exact pathophysiology remains unknown. Here, we present a case of a 53-year-old male with a history of chronic pancreatitis who presented with lower abdominal pain found to have osteolytic pelvic lesions considered to be panniculitis secondary to pancreatitis. Our patient provided an interesting clinical picture given his alcohol use disorder, and lytic lesions which lead the team initially towards a malignant etiology of panniculitis such as myeloma; however, given his negative studies, it was presumed his panniculitis was derived from his chronic pancreatitis. Overall, additional literature is warranted regarding the extensive workup of lytic bone lesions that present in patients who have acute vs chronic pancreatitis.

## Introduction

Panniculitis, inflammation of subcutaneous adipose tissue, was first identified in association with pancreatic disease in the late 1800s [1]. Since then, there have been reports of panniculitis secondary to various types of pancreatic disease including but not limited to, acute pancreatitis, chronic pancreatitis, pancreatic pseudocyst, and pancreatic carcinoma [2]. This phenomenon is very rare with only 0.3%-3% of patients with underlying pancreatic disease developing panniculitis [2]. Here, we present a case of a male with a history of alcohol use disorder and chronic pancreatitis, who was diagnosed with panniculitis secondary to pancreatitis.

## Case presentation

A 53-year-old male with a history of type 2 diabetes mellitus, hypertension, dyslipidemia, hypothyroidism, major depressive disorder, alcohol use disorder, and chronic pancreatitis presented with lower abdominal pain and was found to have acute descending colon diverticulitis with an abscess, with an incidental finding of lytic pelvic lesions, who was subsequently worked up for myeloma and was treated with fluid and antibiotics.

**Fig. 1:**
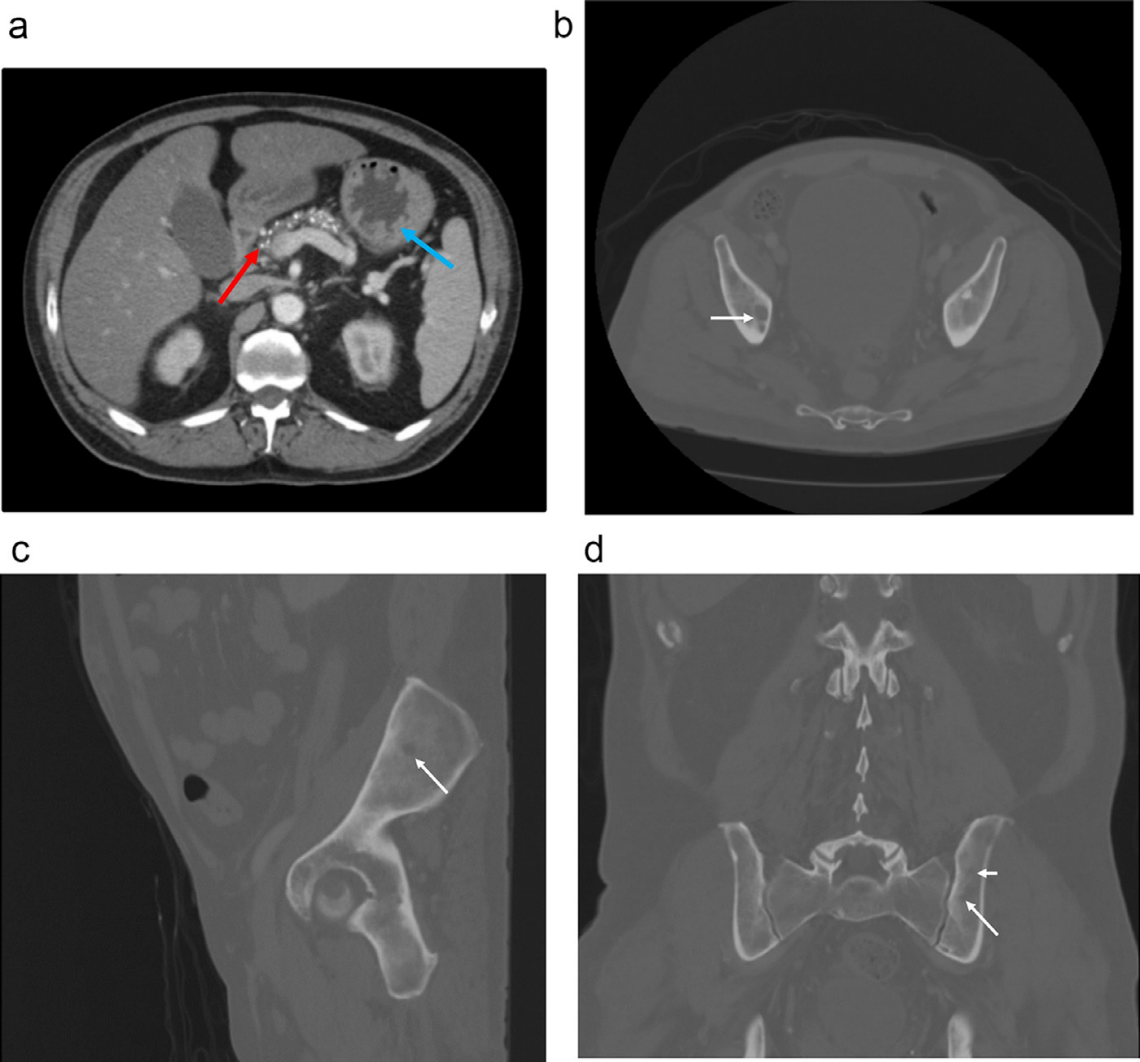
Computed tomography of the abdomen and pelvis notable for acute diverticulitis of descending colon with abscess (A, blue arrow), liver parenchymal disease, chronic pancreatitis (A, red arrow), and incidental lytic lesions (B-D, white arrows).

Within the past 2 years, the patient had 2 prior episodes of acute pancreatitis and endorsed daily alcohol consumption. Computed tomography of the abdomen and pelvis demonstrated acute diverticulitis of the descending colon with associated abscess, liver parenchymal disease, chronic pancreatitis, and an incidental finding of lytic pelvic lesions and a posterior left iliac bone/left acetabular cyst sclerotic lesion (Fig. 1).

**Fig. 2:**
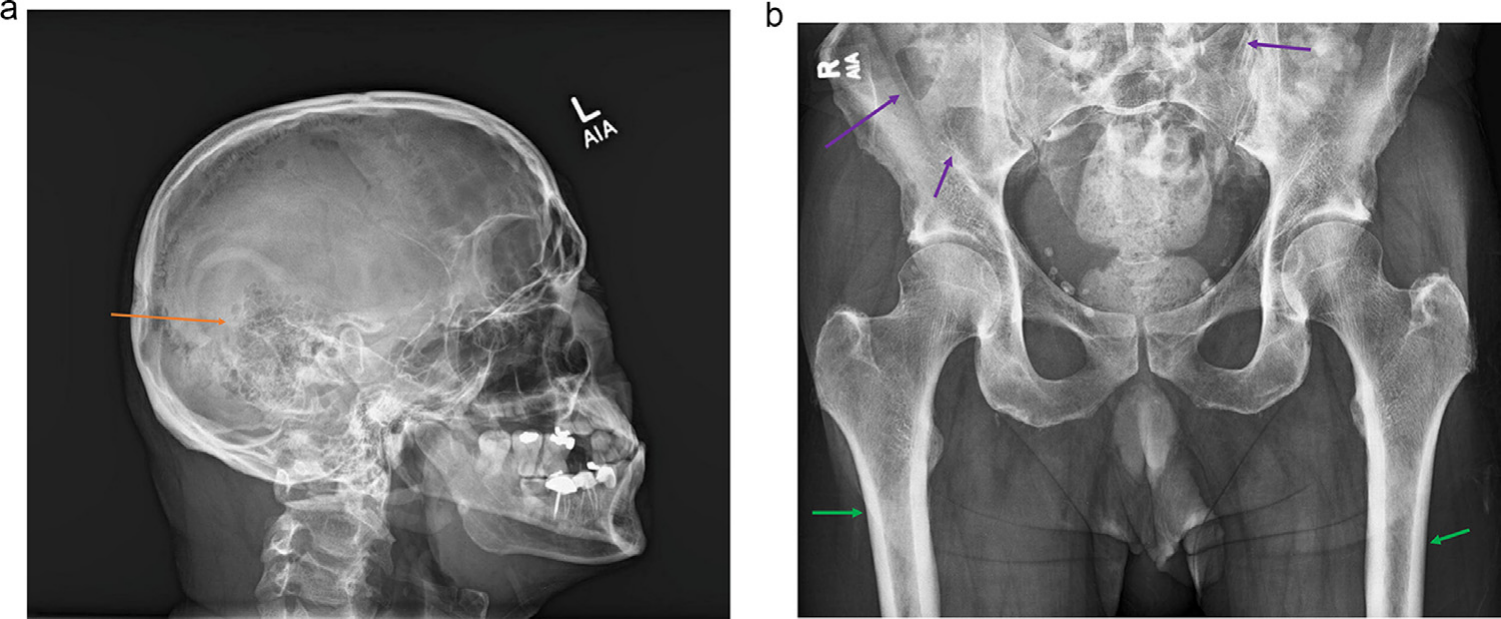
Skeletal Survey with (A) notable for 6mm lucent lesion within the bony calvarium on the lateral view (orange arrows) and (B) notable for questionable re-demonstration of lucent lesions within the bilateral pelvic bones vs overlying bowel gas (purple arrows) as well as cortically based sclerotic lesion along the lateral distal femur that is nonspecific (green arrows).

The patient underwent serologic workup for multiple myeloma, which was negative. A skeletal survey demonstrated lucent lesions within the bony calvarium and questionable re-demonstration of lucent lesions in the bilateral pelvic bones and a cortically based sclerotic lesion along the distal femur, with chronic rib fracture of the right 11th posterior rib (Figs. 2A and B).

Given his findings, the patient's osteolytic pelvic lesions were considered to be panniculitis secondary to pancreatitis, and much less likely secondary to a malignant process. The patient was treated with Piperacillin-Tazobactam for diverticular abscess and intravenous fluids for his pancreatitis and he was discharged home following clinical improvement and resolution of his diverticular abscess on repeat imaging.

## Discussion

The pathogenesis behind panniculitis secondary to pancreatitis is not well described; however, it is thought to be a result of a systemic release of pancreatic holoenzymes, which in turn promote vascular damage and increased permeability [3]. Trypsin plays an important part by increasing the microvascular permeability which promotes the hydrolysis of adipose tissue into free fatty acids and glycerol by lipase and amylase [4]. This mechanism described is not entirely unyielding in that when incubated with adipose, pancreatic enzymes have not been shown to cause fat necrosis in vivo [5]. Another fault of the mechanism described includes reports of pancreatic panniculitis without an increase in pancreatic enzyme serum levels [2].

Pancreatic panniculitis may present clinically in a variety of ways. Variable cutaneous lesions have been described in the literature and may even precede the diagnosis of pancreatic disease in up to 45% of cases [2]. A complication of pancreatic panniculitis is pleural effusion which can indicate a poor prognosis [6]. The Schmid triad is also a poor prognostic factor, and it is described as panniculitis, eosinophilia, and polyarthritis in association with pancreatic disease [2]. Secondary infection due to pancreatic panniculitis is an important complication to consider [7].

Then there is Pancreatitis, panniculitis and polyarthritis (PPP) syndrome, where the third, “P,” or polyarthritis is thought to be a result of periarticular fat pad necrosis [2]. One report finds that in 24 documented cases of PPP most patients were men (70.8% men, 29.2% women), and the average age was 46.6 years [8]. Arthritic disease is relatively prevalent when compared to the other manifestations of pancreatic panniculitis for it is described in 54%-88% of patients who have fat necrosis in conjunction with pancreatic disease [4]. Although any joint can be affected, the ankle, knee, metacarpophalangeal, and knee joints are most commonly involved [9].

Bone harbors marrow which is composed mostly of adipose tissue, 40% of red marrow, and 95% of yellow marrow, which leaves it susceptible to hydrolysis by the pancreatic enzymes [4,10]. The ensuing osteonecrosis is thought to be the result of infarction, described by 2 postulated mechanisms [8]. First, edema and inflammation secondary to intramedullary fat necrosis results in an increase in intraosseous pressure, which in turn, causes decreased perfusion following compression of arterioles and venules [8]. Secondly, the circulating pancreatic enzymes damage the vascular endothelium leading to thrombosis and eventual ischemia [8]. These osseous lesions are most found in the long bones distal to the knee and elbow, and there is typically no involvement of the epiphyses [8]. In our patient, these lesions were observed in both the pelvic bones and the distal femur. The presence of a lesion in the pelvis was elusive in the sense that this is not commonly observed in cases of osteonecrosis secondary to pancreatitis yet is commonly seen with multiple myeloma [11].

Making the diagnosis of PPP and pancreatic panniculitis begins with diagnosing the underlying pancreatic pathology. In the case of pancreatitis, this involves the evaluation of serum amylase and lipase, where amylase cannot be used alone, and lipase is preferred [12]. Abdominal imaging with contrast-enhanced computed tomography or magnetic resonance imaging is recommended in patients whose condition fails to improve within 2-3 days of the onset of symptoms to confirm the diagnosis and assess for any local complications [12]. The capability of the magnetic resonance imaging to detect early intraosseous fat necrosis and edema may allow for an earlier diagnosis when compared to other imaging modalities [8]. Histologic examination of the subcutaneous tissue involved in panniculitis will reveal lobar fat necrosis with characteristic “ghost cells” which are adipocytes that have lost their nucleus yet maintain peripheral outline [2].

The treatment of pancreatic panniculitis and PPP syndrome involves resolving the underlying pathology [9]. There is still no medication that has been effective in treating pancreatitis, so treatment is mostly supportive with intravenous hydration and analgesia in cases of intolerable pain [12]. Octreotide, which is thought to reduce pancreatic secretion, and plasmapheresis have been used in adjunctive therapy in previous cases [9]. Nonsteroidal anti-inflammatory drugs and corticosteroids can be used to manage the symptoms associated with arthritis and cutaneous manifestations, but they have not shown evidence that they decrease the duration of the disease [9]. The prognosis of pancreatic panniculitis greatly depends on the underlying pathology, in cases of acute pancreatitis the panniculitis is likely to resolve with treatment of pancreatitis, while in cases of panniculitis associated with pancreatic carcinoma is much more difficult to treat with a mortality rate near 100% [2].

## Conclusion

In summary, panniculitis has rarely been demonstrated to be secondary to pancreatitis [2]. Pathogenesis of this mechanism may be secondary to pancreatic enzymatic release which may then in turn hydrolyze adipose tissue [4]. Clinical presentation varies; however, patients may experience complications such as pleural effusions, eosinophilia, panniculitis, or polyarthritis [2,6]. A histologic examination may demonstrate lobar fat necrosis with ghost cells [2]. Treatment involves treating the underlying pathology and supportive care [9,12]. Additional research is warranted in further identifying diagnostic and treatment modalities for this rare disease process.

## Ethics statement

An ethical review is not necessary, because this is a case report.

## Previous presentation

This case report was presented as a poster presentation at the American College of Gastroenterology conference in 2022.

## Patient consent

Informed consent for publication of their case was obtained from the patient.

## Authors' contributions

Anas Mahmoud and Isaac Soliman are the article guarantors. Anas Mahmoud, Isaac Soliman, and Brooke Kania performed the literature review and wrote the manuscript. All authors assisted in the collection of the patient's clinical data. All authors took part in the medical management of the patient and edited the final manuscript for submission.
